# cDNA Library Enrichment of Full Length Transcripts for SMRT Long Read Sequencing

**DOI:** 10.1371/journal.pone.0157779

**Published:** 2016-06-21

**Authors:** Maria Cartolano, Bruno Huettel, Benjamin Hartwig, Richard Reinhardt, Korbinian Schneeberger

**Affiliations:** 1 Department of Plant Developmental Biology, Max Planck Institute for Plant Breeding Research, Cologne, Germany; 2 Max Planck-Genome-centre Cologne, Max Planck Institute for Plant Breeding Research, Cologne, Germany; University of California, Los Angeles, UNITED STATES

## Abstract

The utility of genome assemblies does not only rely on the quality of the assembled genome sequence, but also on the quality of the gene annotations. The Pacific Biosciences Iso-Seq technology is a powerful support for accurate eukaryotic gene model annotation as it allows for direct readout of full-length cDNA sequences without the need for noisy short read-based transcript assembly. We propose the implementation of the TeloPrime Full Length cDNA Amplification kit to the Pacific Biosciences Iso-Seq technology in order to enrich for genuine full-length transcripts in the cDNA libraries. We provide evidence that TeloPrime outperforms the commonly used SMARTer PCR cDNA Synthesis Kit in identifying transcription start and end sites in *Arabidopsis thaliana*. Furthermore, we show that TeloPrime-based Pacific Biosciences Iso-Seq can be successfully applied to the polyploid genome of bread wheat (*Triticum aestivum*) not only to efficiently annotate gene models, but also to identify novel transcription sites, gene homeologs, splicing isoforms and previously unidentified gene loci.

## Introduction

Complete and accurate gene annotations are essential to make full use of the underlying genome assemblies. The development of high-throughput short read RNA sequencing (or RNA-seq) technologies has greatly advanced the study of genomes by enabling both the improvement of genome annotations and the study of organisms for which reference genomes are not yet available [1–3]. However, reference-free as well as reference-based transcript assemblies of short reads are challenging [1] and often do not match experimentally validated gene models [4]. In fact, annotation of complex genomes, such as those of plant species with agricultural relevance, is still not leading to optimal results. In addition to imperfect gene model reconstruction, the incompleteness of the underlying genome assembly is complicating gene annotations.

In 2014, the International Wheat Genome Consortium (IWGC) released a chromosome arm-based draft sequence of 10.2 GB of the bread wheat landrace ‘Chinese Spring’ (Chromosome Survey Sequence or CSS), 4.5 GB of which have been ordered in chromosomes pseudo-molecules using the POPSEQ technology [5–8]. Gene annotation using full-length cDNAs and RNA-seq data from five tissues and three developmental stages enabled the prediction of 124,201 high confidence gene loci (i.e. genes with homology to related grasses) and 325,147 splicing isoforms [8]. Even though, the homeologous loci are likely well defined due to the sequencing of the individual chromosome arms, the completeness of the wheat genome annotation relies upon the length and the quality of the individual assemblies [9]. Furthermore, given the high nucleotide identity observed between coding sequences of homeologous loci, it is likely that unique gene assignment of short RNA reads is yet not fully accurate.

Pacific Biosciences’ (PacBio) Single Molecule Real Time Sequencing (SMRT) promises to overcome some of the difficulties presented by the complexity of the wheat transcriptome. While producing long reads it enables identification of bona fide mRNA transcripts and novel splicing isoforms [10] and has already been applied for gene prediction improvement of genomes [11–16]. However, sequencing full-length transcripts still represents a challenge due to the presence of truncated transcripts from RNA degradation, mechanical shearing and incomplete cDNA synthesis in the cDNA libraries [12].

To enrich for real full-length transcripts we implemented the TeloPrime Full-Length cDNA Amplification Kit, which selectively synthesises cDNA molecules from mRNAs carrying a 5’ cap, to the standard Pacific Biosciences cDNA library preparation protocol. The TeloPrime-based protocol outperformed the standard SMARTer PCR cDNA Synthesis kit in the enrichment for SMRT reads containing genuine transcription start sites when tested on the genetic model system *A*. *thaliana* [17,18]. We also applied this approach to *T*. *aestivum* providing an example of how full-length transcripts-enriched long read SMRT sequencing could improve the annotation of complex polyploid genomes.

## Materials and Methods

### Plant material and growth conditions

*A*. *thaliana* (Col-0) plants were grown in soil pots. To avoid RNA contamination, *T*. *aestivum* (Chinese Spring) seeds were sterilised in a sodium hypoclorite solution (Roth, Germany) for seven minutes and transferred in glass beakers containing a sucrose enriched, agar based, germination medium (GM medium) prepared as described in [19]. *A*. *thaliana* and *T*. *aestivum* were grown in conditions of 16 hours light and 8 hours darkness.

### RNA isolation

Total RNA was isolated from inflorescence tissue of *A*. *thaliana* and leaf tips (9 days after germination) of *T*. *aestivum* with RNeasy Plus Micro Kit (Qiagen, The Netherlands). RNA was quantified by spectrophotometry (Nanodrop, Thermo Scientific, USA) and quality assessed with a 2100 Agilent Bioanalyser (RNA Nanochip, Agilent Technologies, Germany).

### cDNA PCR library preparation with template switch method

First strand cDNA synthesis was performed using the SMARTer PCR cDNA Synthesis Kit (Clontech Laboratories, USA) from 1 μg of total RNA input according to manufacturer’s instructions. The first-strand cDNA synthesis is primed by 3’ SMART CDS Primer II A at the 3’ poly A stretch. The SMARTScribe MMLV Reverse Transcriptase catalyses this reaction and, when it reaches the 5’ end of the mRNA, adds a few non-template nucleotides (mostly Cs) at the 3’ end of the cDNA. In presence of the 5’ PCR Primer II A, which anneals to the newly added non-template nucleotides, the SMARTScribe RT switches template and synthesises until the end of the Primer II A oligo [20].

The first strand cDNA:RNA hybrid molecules were 5-fold diluted and PCR amplification with the KAPA HiFi polymerase (KAPA HiFi PCR kit, Peqlab, Germany) was performed, column purified (Qiagen PCR purification kit) and inspected (2100 Agilent Bioanalyzer, D12000) to identify the optimal cycle number for large scale PCR. The optimal cycle was defined by absence of PCR artifact pattern in low and high molecular weight range due to over-cycling [21]. Large scale PCR with the optimal cycle number (17 for *A*. *thaliana*) was performed to achieve sufficient cDNA for PacBio library preparation.

### cDNA PCR library preparation with cap-dependent linker ligation method

The TeloPrime Full-Length cDNA Amplification Kit (Lexogen, Austria) was used for generating full-length cDNA from 1 μg of total RNA. First strand cDNA synthesis is initiated by a 3’ oligo-dT anchoring primer (RP: 5’-TCTCAGGCGTTTTTTTTTTTTTTTTTT-3‘) and reverse transcription. The cDNA:RNA hybrid molecules were column purified and ligated to the double-stranded linker (cap-dependent linker ligation, CDLL) carrying a 5’C overhang, thus allowing base pairing with the G nucleotide of the 5’ mRNA CAP. Ligation products were again column purified and the resulting eluted fragments were converted to full-length double-stranded cDNA by second strand cDNA synthesis.

The full-length double-stranded cDNAs were first amplified in a qPCR reaction using 3’ and 5’ end-specific primers (RP and FP: 5'–TGGATTGATATGTAATACGACTCACTATAG–3') to determine the cycle number for the large scale PCR. SYBR Green I (Invitrogen,USA) was added to a final concentration of 0.1x in the qPCR reaction with a total of 40 cycles. qPCR results were evaluated to determine the fluorescence value where the fluorescence has 80% of the maximum. The determined cycle number (17 for *A*. *thaliana*, 17 for *T*. *aestivum*) was applied for large scale PCR in the absence of SYBR Green I.

### PacBio library preparation

The large scale amplified cDNAs obtained with both synthesis protocols were pooled and column purified (Qiagen PCR Purification Kit, Qiagen, The Netherlands), run on a 1% agarose gel and three separate size ranges were fractionated: 1–2 kb, 2–3 kb, and over 3 kb. Each size fraction was extracted from the gel (Qiagen Gel Extraction Kit, Qiagen, The Netherlands), purified and amplified for additional eight PCR cycles with SMARTer or TeloPrime specific primer pairs, respectively. PCR products were again pooled and column purified (Qiagen PCR Purification Kit, Qiagen, Netherlands). Single Molecule Real Time (SMRT) bell libraries were prepared as recommended by Pacific Biosciences (Palo Alto, U.S.A). SMRT bell templates were bound to polymerase using the DNA polymerase binding kit P6 v2 primers.

### PacBio sequencing

Polymerase-template complexes were bound to magnetic beads using the Magbead Binding Kit and sequencing was carried out on the PacBio RS II sequencer using C4 sequencing reagents with movie lengths of 240 min (*A*. *thaliana*) or 360 min (*T*. *aestivum*) on two SMRT cells for each experiment.

### Data analysis

Raw reads were initially assembled using the Pacific Biosciences' SMRT analysis software version 2.3.0 [11]. The polymerase reads were partitioned into subreads. Read of Inserts (ROI) were generated using the default number of polymerase full passes. The Iso-Seq *classify* tool was then used to separate the ROIs in full length and non-full length, non-chimeric, reads. Full-length reads were defined as containing both 5’ and 3’ cDNA primers. The Iso-Seq *cluster* tool was then used to cluster all the full-length reads derived from the same transcript isoform using a Minimum Quiver Accuracy setting > = 0.99. In the last stage, the non-full-length reads were used to polish the consensus sequences produced by the Iso-Seq *cluster* tool using the *Quiver* algorithm [11].

High Quiver (HQ) polished reads were aligned to the *A*. *thaliana* and *T*. *aestivum* reference genomes using GMAP (2015-07-23) [22] with default settings. *A*. *thaliana* v10 genome assembly was downloaded from TAIR (www.arabidopsis.org) [18]. *T*. *aestivum* genome assembly was downloaded from Ensembl plants, gene build version 2.2 (http://plants.ensembl.org/Triticum_aestivum/Info/Annotation/#assembly). The chromosome arm assemblies were downloaded from PGSB’s PlantsDB (http://pgsb.helmholtz-muenchen.de/plant/wheat/iwgsc/index.jsp) [8]. HQ reads aligning to their genomic targets with > = 90% nucleotide identity were selected for further analysis. In order to retrieve full-length (FL HQ) reads, the alignment coordinates were compared to the genome reference annotations. HQ reads were categorized as FL HQ when equal or larger in length than their respective targets. Wheat HQ reads were also aligned against the nr database using the BLASTX algorithm with an E-value cutoff of 1e-03. Blast2go v3.1 was used with default settings for functional annotation of the HQ reads not aligning to the current Ensembl reference genome. Identification of novel wheat splicing isoforms was performed by aligning the HQ reads to the CSS assembly [8]. Alignments were performed with GMAPL (2015-07-23) [22] with default settings. Cuffcompare v2.2.1 was used to compare the HQ reads to the CSS assembly [23]. To identify HQ reads aligning to homeologous loci, the GMAPL coordinates were compared to the list of High Confidence homeologous loci downloaded at ftp://ftpmips.helmholtz-muenchen.de/plants/wheat/IWGSC/genePrediction_v2.2/ta_IWGSC_MIPSv2.2_HighConf_REPR_BBH-TRIPLETS_2014Jul18.tab.

## Results and Discussion

### 5’ capped transcript enrichment using the TeloPrime Full-Length cDNA amplification kit

**Fig 1:**
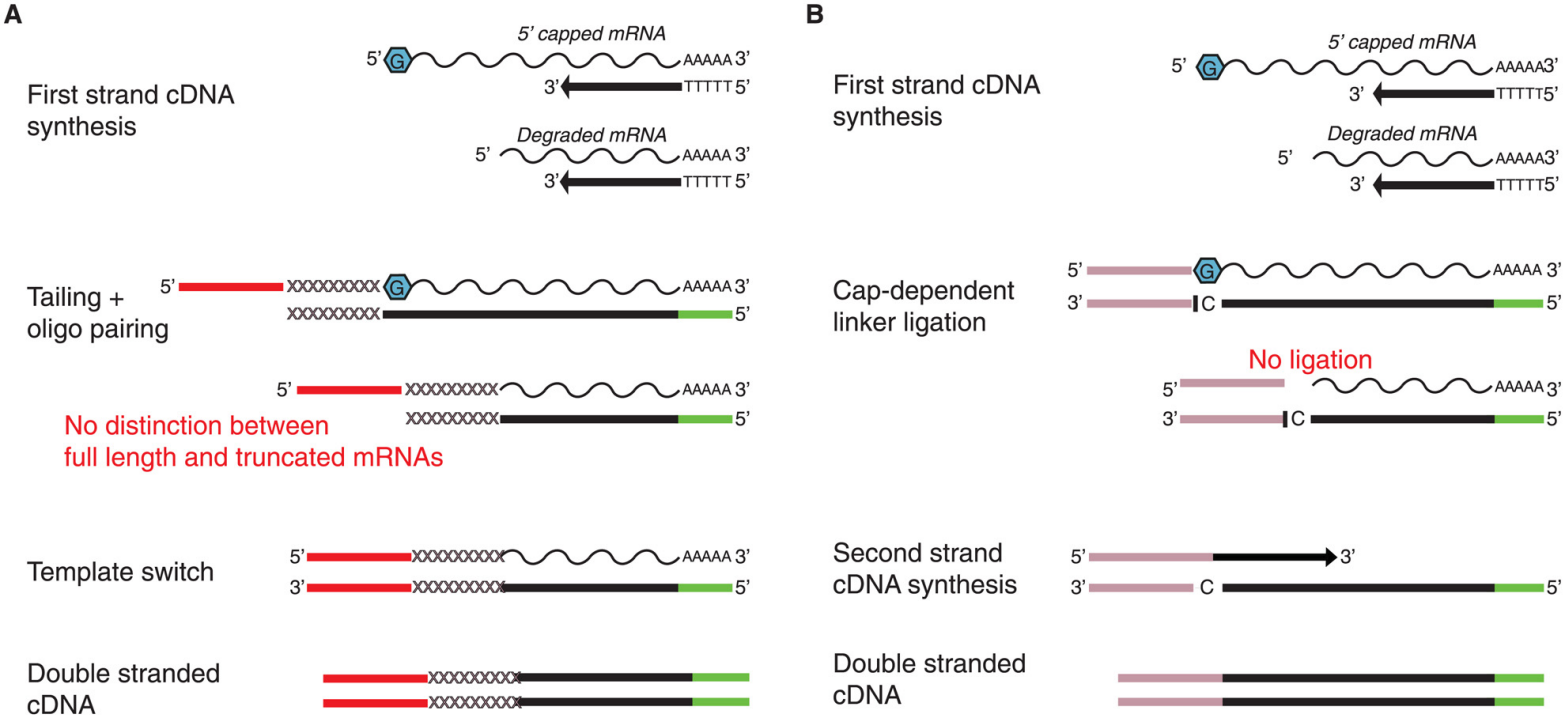
Schematic diagram of the TeloPrime and SMARTer cDNA synthesis technologies. (A) SMARTer does not distinguish between truncated and full-length transcripts. (B) TeloPrime enriches for full-length transcripts by affinity of the 5’ adapter to the 5’ cap structure of full-length mRNAs during cDNA synthesis. Red and pink rectangular shapes are the 5’ adapters provided by each kit, respectively. Green rectangular boxes are the oligos priming cDNA synthesis of poly-A transcripts.

PacBio currently employs the SMARTer technology, which takes advantage of the “template switching” effect mediated by the MMLV Reverse Transcriptase, to limit the occurrence of incomplete cDNA synthesis. However, the SMARTer protocol does not distinguish between full-length and truncated transcripts (Fig 1A), thus hampering long read sequencing of full-length mRNAs. We tested whether the implementation of the TeloPrime technology, which selectively filters for 5’ capped mRNAs during cDNA synthesis, can sensibly improve long read sequencing of full-length transcripts (Fig 1B).

We compared the performance of the two protocols by using *A*. *thaliana*, as it features a genome annotation of highest quality as it has been periodically updated over the past years [17,18]. Each experiment was performed on two SMRT cells and yielded a total of 24,135 TeloPrime and 30,192 SMARTer HQ reads, which aligned to the *A*. *thaliana* reference genome with > = 90% nucleotide identity (Table 1 and see Table A in S1 File). The degree of residual redundancy in both datasets was extremely low. Clustering both read sets (while allowing for 100 and 5 bp differences at 5’ and 3’ ends of the reads) revealed between as little as 0.08% to 0.75% of putatively redundant reads per data set.

**Table 1:** *Arabidopsis thaliana* PacBio Iso-Seq output summary. cDNA was synthesised with Lexogen TeloPrime Full-Length amplification kit or the Clontech SMARTer PCR cDNA Synthesis Kit and then split into three size fractions (1–2, 2–3 and 3–6 kb), respectively. From left to right: number of transcript isoform clusters (HQ reads) assembled using the PacBio Iso-Seq pipeline; number of HQ reads aligning to the *A*. *thaliana* genome with a sequence identity > = 90%; percentage of full length HQ reads (FL) defined as the cumulative number of reads equal or larger in length than their respective target.

cDNA library	Size fraction (kb)	HQ reads	HQ reads (> = 90%)	% FL HQ reads
TeloPrime	1–2	7,679	7,443	4
SMARTer	1–2	1,9449	18,223	2.8
TeloPrime	2–3	15,119	14,430	7.2
SMARTer	2–3	14,054	7,699	7.6
TeloPrime	3–6	4,139	2,262	13
SMARTer	3–6	7,080	4,270	10

**Fig 2:**
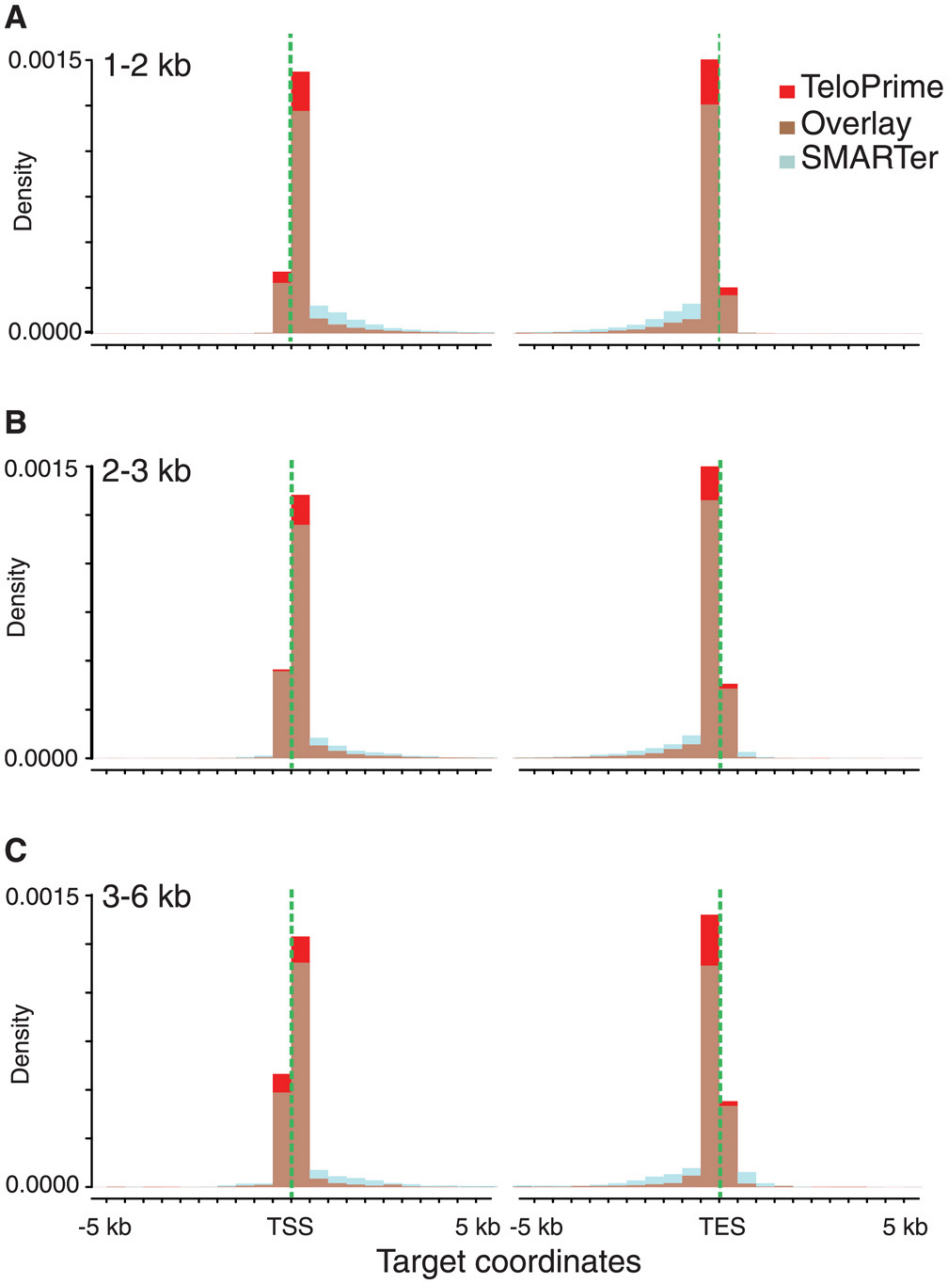
Transcription start and end site enrichment using the TeloPrime Full-Length cDNA amplification kit (Lexogen). (A-C) Superimposed density plots of the PacBio Iso-Seq alignment coordinates against the 5’ and 3’ ends of their targets. TeloPrime cDNA libraries (red bars), SMARTer cDNA libraries (light blue bars), overlay of the two protocols (brown bars). (A) 1–2 kb size fraction; (B) 2–3 kb size fraction; (C) 3–6 kb size fraction. 10 kb around the annotated gene start and end coordinates are shown on the x-axis. Vertical green dashed lines highlight annotated target start and end sites.

As TeloPrime selectively synthesises cDNA from 5’ capped (full length) mRNA structures, TeloPrime HQ reads should be enriched for alignments starting near the annotated target transcription start site (TSS). Indeed, as compared to the SMARTer reads, a significantly higher proportion of TeloPrime HQ reads aligned to the TSS, although alignments starting downstream of the annotated TSS (revealing presumably not-full-length transcripts) could be observed for both experiments (Fig 2; Mann-Whitney test for each size fraction: 1–2 kb, p-value < 2.2e-16; 2–3 kb, p-value = 2.93e-12; 3–6 kb, p-value = 9.607e-15). Interestingly, read alignments starting upstream of annotated TSS were also preferentially TeloPrime HQ reads, highlighting the potential of our proposed protocol to improve gene start prediction even for highly curated genomes.

OligodT priming is employed by both SMARTer and TeloPrime to synthesise first strand cDNAs from the 3’ of polyA mRNAs. Therefore, we expected 3’ gene end annotations to be similarly represented in both experiments. However, as for the 5’ ends, the TeloPrime HQ reads showed more alignments reaching to the transcription end sites (TES) as compared to the SMARTer reads (Fig 2; Mann-Whitney test for each size fraction: 1–2 kb, p-value < 2.2e-16; 2–3 kb, p-value = 2.73e-16; 3–6 kb, p-value = 1.554e-05). It is possible that 5’ capped mRNAs are less prone to be targeted by the RNA surveillance mechanism and, therefore, bona fide full-length transcripts are more likely to be represented in the TeloPrime cDNA libraries.

### Case study: Sequencing of wheat TeloPrime cDNA libraries for genome curation

*T*. *aestivum* genome assembly has seen a dramatic improvement in the past five years [7–9,24,25]. However, great challenges are still ahead towards the release of an accurate genomic annotation of this hexaploid species. To test whether the TeloPrime-based PacBio Iso-Seq SMRT sequencing can sensibly help improving the prediction of wheat gene models, we ran a small experiment on mRNA extracted from wheat leaf tips. Similarly to the *A*. *thaliana* experiment, the cDNA was split in 1–2, 2–3 and 3–6 kb size fractions and each fraction was ran on two SMRT cells yielding a total of 370,887 ROIs and 25,651 HQ reads (see Tables B and C in S1 File). The HQ reads were aligned against the current Ensembl reference genome using GMAP and alignments were further filtered for 90% nucleotide identity (see Table B in S1 File). In summary, 16,372 (64%) HQ reads aligned unambiguously to the wheat genomes, whereas as little as 504 (2%) retrieved multiple alignments (and were therefore discarded from further analyses). However, the entire remaining 8,775 (34%) HQ reads could not be reliably aligned against the genome at all.

**Fig 3:**
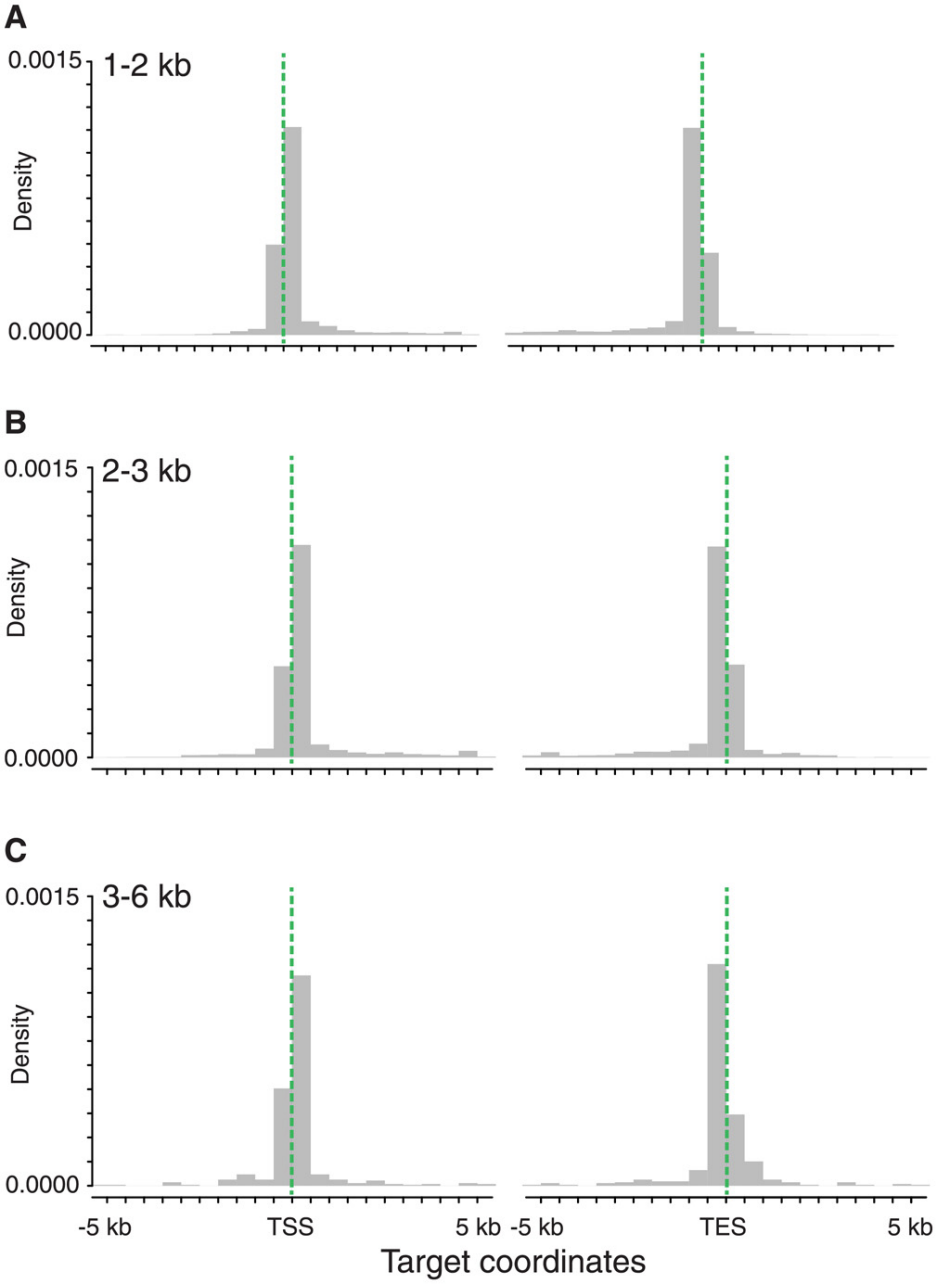
Improved resolution of transcription start and end sites in *T*. *aestivum*. Density plot of PacBio full-length cDNA read alignment ends within 10 kb around the target start and end coordinates are shown for each size fraction; (A) 1–2 kb size fraction; (B) 2–3 kb size fraction; (C) 3–6 kb size fraction. Vertical green dashed lines highlight target start and end sites.

Of the 12,869 HQ reads that uniquely aligned against genes and pseudo-genes, 1,961 HQ reads (15%) were equal or larger in size than their corresponding targets (Table 2) revealing putative novel TSS and TES (Fig 3). This much larger fraction of reads revealing new TSS and TES information, as compared to the same fraction in the *A*. *thaliana* analysis (5%), underlines the possibilities to improve the current wheat annotation by the TeloPrime-based PacBio Iso-Seq for full-length gene annotation.

**Table 2:** Genomic regions detected by the wheat HQ reads. The number of HQ reads aligning to each genome and scaffold. The last column indicates the number of full length reads (FL, i.e. equal or larger in size than the corresponding target) calculated for long and small coding genes and pseudo-genes. Pt: chloroplast.

Genome	HQ reads	Long coding genes	Small coding genes	Pseudo-genes	Repeat region	Not annotated	FL
A	3406	2716	1	-	308	381	92
B	6057	4529	1	107	709	711	1603
D	3663	2952	-	-	300	411	94
Pt	3	2	-	-	-	1	2
Contigs	3243	2548	-	13	231	451	170

Intriguingly, 1,955 (1,512) HQ reads aligned to regions not annotated as genes in the current Ensembl reference genome (CSS assembly) (Table 2) and only 110 (6%) of them could be reliably aligned to the sequences of the PGSB Repeat Element Database (PGBS–Redat) [26], excluding the possibility that most of these loci relate to transposable elements, which were excluded from final gene annotations. Though parts of this might also result from wrong alignments or random transcription, it seems likely that a substantial portion provides evidence for genes that so far have not been annotated despite their presence in the assembly (see Tables D-H in S1 File).

**Fig 4:**
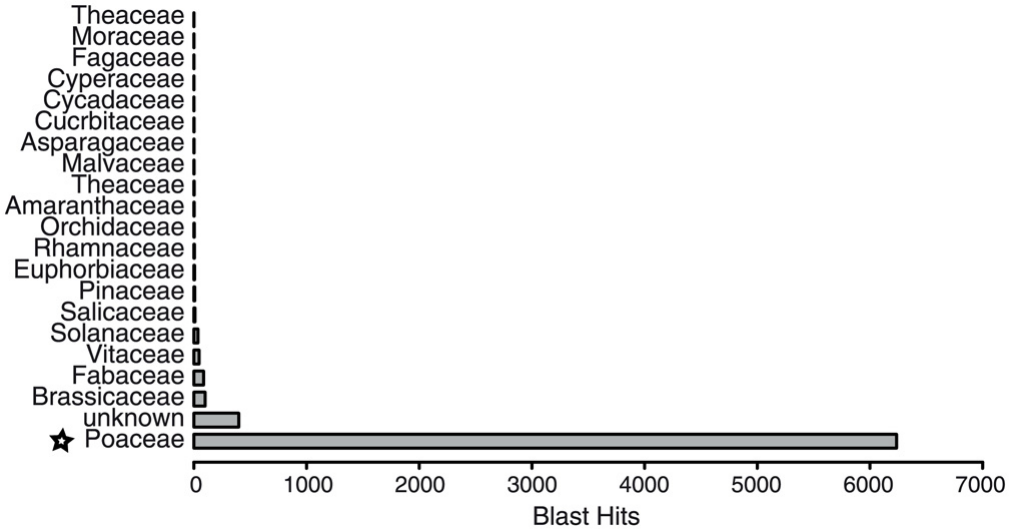
Best blast hit distribution against the nr database using HQ reads not aligning to the *T*. *aestivum* Ensembl genome assembly.

To further estimate the amount of genes that are not even present in the assembly, we aligned the 8,775 HQ reads that could not be aligned against the reference sequence against the nr database using BLASTX. While 1,823 (21%) HQ reads are likely to represent low quality reads or contamination (e.g. no or no plant specific Blast hits), 6,952 (79%) showed homology to plant protein sequences (e-value cut-off < 1E-03), while only around 13% of them showed additional similarity to transposable elements (again as estimated with alignments against PGBS–Redat). Interestingly, 90% of the Blast hits against the nr database are homologous to protein sequences derived from close relatives of *T*. *aestivum* (i.e. members of the *Poaceae* family) suggesting that up to 27% of all HQ reads are likely to represent genes, which are not yet accurately assembled (Fig 4 and see Table I in S1 File). This shows that full-length transcriptome sequencing can immediately and reliably (that is without *de novo* RNA assembly) improve our knowledge on genes even in species without complete genome assemblies.

In addition to gene co-ordinates, gene annotations try to describe the complement of diverse transcripts derived from individual loci that result from alternative splicing. As the underlying gene sequence is identical, transcript evidence is absolutely crucial for correct annotation of diverse isoforms. To estimate the presence of potentially novel splicing isoforms in our data we aligned the HQ reads against a different genome assembly, *T*. *aestivum* CSS assembly [8], as it contains a more comprehensive list of splicing isoforms. After filtering for hits with > = 90% nucleotide identity, we obtained 15,592 unique alignments that were screened for novel isoforms using CuffCompare [23]. We found 5,920 isoforms already present in the annotation (corresponding to 5,562 distinct genes). Moreover 2,008 reads revealed novel isoforms (corresponding to 2,004 distinct genes), which have not been included in the gene annotation so far (see Tables J and K in S1 File).

Finally, we estimated the feasibility of the TeloPrime-based PacBio sequencing to distinguish between homeologous loci, again using the second set of alignments against the CSS assembly. Only 996 (6%) out of 16,372 HQ reads were aligned to more than one homeologous locus. This number was further reduced to five HQ reads when filtering was applied based on 90% nucleotide identity and best alignment match (i.e. difference between HQ read length and alignment length). This surprisingly small number strongly suggests that assignments of transcripts to the correct homeologous locus is greatly simplified by PacBio Iso-Seq sequencing paving the way for highly accurate homeologous-specific transcripts profiling.

## Conclusion

Despite the small size of our experiment, we showed that TeloPrime-based PacBio Iso-Seq improves state-of-the-art library preparation and that a considerable amount of novel information can be gained by employing this protocol to the hexaploid wheat genome. Given that around half of the extant angiosperms are polyploid we believe that the implementation of the TeloPrime-based PacBio sequencing could greatly facilitate current and future efforts in genome annotation [9].

## Availability of Supporting Data

All PacBio read data will be made public upon publication of the manuscript in Genbank (BioProject No.: PRJNA306427; BioSamples No: SAMN04456597 till SAMN04456606).

## Supporting Information

S1 File Supplementary tables. (XLSX)
